# Different names, different discrimination? How perceptions of names can explain rental discrimination

**DOI:** 10.3389/fsoc.2023.1125384

**Published:** 2023-03-02

**Authors:** Billie Martiniello, Pieter-Paul Verhaeghe

**Affiliations:** Department of Sociology, Vrije Universiteit Brussel (VUB), Brussels, Belgium

**Keywords:** discrimination, names, housing market, signals, ethnicity, correspondence tests, field experiment

## Abstract

**Introduction:**

The aim of this research is to look into how signals carried by names can contribute to the explanation of why rental discrimination is measured. Ethnic discrimination on the rental housing market is a widespread phenomenon that is increasingly captured in an objective way by means of correspondence studies. This methodology assumes that the trigger for the measured discriminatory behavior is the signal of ethnic origin in names. Nonetheless, previous research found that names also contain other signals such as religiosity, social class and educational level.

**Method:**

The current study relates data of 3.753 correspondence tests on the Flemish rental housing market in Belgium to survey data about the perception of the used names in the correspondence tests in terms of ethnic origin, gender, religiosity, social class and educational level. By doing so, we can set a step in the direction of why discrimination is measured.

**Results and discussion:**

We find that realtors discriminate based on the perception of a name as religious, which we describe as religious taste-based discrimination. Besides, we also find indications for statistical discrimination. For landlords, the perception of a name as non-European increases discrimination, indicating ethnic taste-based discrimination.

## 1. Introduction

Already many studies have shown persistent patterns of discrimination against ethnic-cultural minority groups in both labor and housing markets (Zschirnt and Ruedin, 2016; Flage, 2018; Auspurg et al., 2019; Quillian and Midtbøen, 2021). Many of these studies rely on field experiments, such as correspondence or audit tests, to measure discriminatory behavior in the real world (Gaddis, 2018; Verhaeghe, 2022). In these tests, two candidates apply for real job vacancies or rental advertisements. Both candidates are as similar as possible and only differ with respect to their ethnic origin. Afterwards, the reactions of recruiters or landlords are compared, and a significant unequal treatment of the candidates is considered as evidence for ethnic discrimination. Because they validly assess adverse behavior in the field within an experimental design with test and control groups, correspondence tests are seen as the “golden standard” to measure discrimination.

Nevertheless, a major limitation of these tests is that many of them only measure levels and patterns of discrimination, but do not examine the mechanisms explaining discrimination (Gaddis, 2019c; Quillian and Midtbøen, 2021). Some correspondence studies aim to investigate the underlying drivers from information given in the advertisement or by varying the level of information given about the candidate, but conclusions remain mixed (Auspurg et al., 2019; Lippens et al., 2022). A promising avenue to further advance discrimination research is, therefore, to combine correspondence test data with other methods of data collection, like data about contextual factors (Gaddis, 2019c). A few studies on rental discrimination already combined correspondence tests with survey data on anti-immigrant attitudes, aggregated at the municipality level (e.g., Carlsson and Eriksson, 2017; Verhaeghe and De Coninck, 2022). This study proposes a different strategy of data triangulation by digging deeper into the perceptions of names used in correspondence tests.

In correspondence studies there is no direct, visual contact between candidates and landlords or realtors, as these direct contacts are much more difficult to control and standardize in the experiment (Verhaeghe, 2022). Therefore, names are used as invisible signals of ethnic origin of candidates (Bertrand and Mullainathan, 2004; Carpusor and Loges, 2006). Names, however, signal more than only ethnic origin (Tuppat and Gerhards, 2021). Previous research has shown that people perceive names also in socioeconomic and religious terms (Gaddis, 2019a,b; Martiniello and Verhaeghe, 2022). Even names from the same ethnic group differ substantially in the social class, educational or religious markers they convey. The aim of this study is to use this sociological variation in the perception of names to further disentangle the different underlying mechanisms of rental discrimination against ethnic minorities. For this goal, we combined data from a thorough survey on the subjective perception of names in terms of ethnic origin, religiosity, social class and educational level with data from 3.753 correspondence tests measuring discrimination against candidates with Moroccan and Polish names on the rental housing market of Belgium. Since private landlords substantially differ from real estate agents in the level and nature of their discriminatory behavior (Flage, 2018), this study distinguishes between both rental suppliers.

The Moroccan and Polish communities in Belgium are interesting to study since they profoundly differ in their migration history and ethno-religious aspects. Large-scale immigration from Morocco started during the golden 1960s, first as “guest laborers” and since the 1970s increasingly under the form of marriage migration and family reunification. In contrast, Polish migration to West-Europe is a more recent phenomenon, that really took off during the nineties after the disintegration of the Soviet-Union. Most Polish migrants came first mainly undocumented (and to a much lesser extent as asylum seekers), but since the enlargement of the European Union in 2004 as regular EU-citizens (Martiniello and Rea, 2012). As the result, the residents of Moroccan origin currently represent 3.2 percent of the Flemish population, whereas people of Polish origin represent 1 percent (each time including both the first and second generation) (Statbel, 2022). While the Moroccan migrants are the biggest non-European ethnic community in Belgium and are predominantly Islamic in their religious beliefs, the Polish are a smaller community and are mainly Catholic. Anti-immigration attitudes in Belgium are worse about migrants from non-European, Muslim countries than about European, and non-Muslim migrants (De Coninck, 2020). In addition, the popular far-right party in the Flemish region especially stigmatizes the non-European migrants from the Maghreb and Turkey, and less the intra-European migrants.

In the next section we briefly elaborate on the main theoretical explanations for discrimination and how these relate to the perceptions of names. Subsequently, we discuss how these perceptions also reflect different ethnic, religious and socioeconomic boundaries. Both theoretical sections result in five hypotheses. Afterwards, we describe the data and methodology of this study. The following section presents the main findings for realtors and private landlords. Finally, we end with a discussion of the implications of these findings for discrimination research.

## 2. Explaining rental discrimination

Although different explanations and theories might be relevant (Pager and Shepherd, 2008), two economic theories are regularly cited to explain rental discrimination (Flage, 2018). Firstly, statistical discrimination relies on the idea of a risk balance, falling back on stereotypes and prejudices in the face of a lack of information about a candidate (Becker, 1957; Arrow, 1971). For the rental market, realtors or private landlords want to assess the capability of candidates to pay rent—among other factors, and thus their socioeconomic status in order to assess their solvability. However, realtors or private landlords often have no or insufficient information about a candidate's socio-economic status in the initial phase of the rental process. In this case, realtors and private landlords might rely on—usually negative—stereotypes and prejudices that are related to the ethnic origin of the candidate, consequently denying or diminishing the opportunity to rent the dwelling (Ross and Turner, 2005). Thus, following this theory, the underlying mechanism of ethnic discrimination is the perceived socioeconomic status of a rental candidate.

Secondly, taste-based discrimination assumes that discriminatory behavior is the result of personal preferences or “(dis)tastes” toward certain ethnic minority groups. Here, a subdivision can be made between agent taste-based and customer taste-based discrimination (Ross and Turner, 2005; Carpusor and Loges, 2006; Verstraete and Verhaeghe, 2020). In the former, the realtor or private landlord holds distastes toward certain ethnic minority groups, leading to rental discrimination. The latter is related to the ethnic distastes of customers, which could be both (potential) neighbors, and owners of the rental property (Yinger, 1986; Hanson and Hawley, 2014; Van der Bracht et al., 2015). Consequently, realtors may assume that ethnic majority customers are unwilling to live next to or rent their dwelling to ethnic minority members. This assumption might translate in discrimination toward ethnic minority rental candidates. Here, the underlying mechanism of ethnic discrimination is the (perceived) ethnic-cultural origin of a rental candidate.

Nonetheless, (Western) Europe is characterized by a rather color-blind ideology, whereby the importance of ethnic origin is downplayed or even ignored (Lewis, 2004; Cretton, 2018). Despite this, ethnic discrimination persists in a more “subtle” way, driven by motives other but related to ethnic origin (Bonilla-Silva, 2003). Strong importance is given to the distinction between historically (Judo-)Christian but secularizing Europe on the one hand and other religions, specifically Islam, on the other (Zolberg and Woon, 1999; Ponce, 2019). A double distinction can thus be made: (1) non-religious vs. religious and (2) Christianism vs. other religions and Islam in particular (Zolberg and Woon, 1999; Phalet et al., 2013). Hence, besides ethnic taste-based discrimination, two additional forms might be added which we will name religious taste-based discrimination and religion-specific taste-based discrimination. The former implies ethnic discrimination driven by distastes toward being (perceived as) religious. The latter refers to ethnic discrimination which arises through distastes toward specific religions. Note that both forms are in practice probably strongly intertwined as, for example, being Muslim is often related to the stereotype of being strongly religiously committed (Iyengar et al., 2013). We disentangle them here for the sake of theoretical clarity.

To analyze which theory—statistical or taste-based discrimination—researchers find support for, additional explicit cues are provided, such as information regarding a person's job, which conveys signals of his or her social class. If statistical discrimination is the main driver, additional information should reduce the risk for realtors and landlords and consequently lower discriminatory behavior (Ross and Turner, 2005; Ahmed and Hammarstedt, 2008; Baldini and Federici, 2011; Auspurg et al., 2017; Horr et al., 2018). Adding information ignores the possibility that research subjects perceive also other signals in names, which may trigger a certain behavior. The latter is referred to as the excludability assumption (Butler and Homola, 2017). However, both in an American (Gaddis, 2019a,b; Crabtree et al., 2022) and European context (Martiniello and Verhaeghe, 2022) names are found to contain, besides ethnic signals, indications of religiosity, social class, immigrant status, and educational level.

It is possible that through correspondence testing ethnic discrimination is measured, which is related to these other signals. Thus, names as signals might contain an explanatory potential. If the measured ethnic discrimination is related to perceived socioeconomic signals, these tests measure ethnic discrimination that is brought about by stereotypes and prejudices and is thus in line with statistical discrimination. Research already found that names are used as an indicator of a person's socioeconomic status when more direct information is lacking (Acolin et al., 2016). If ethnic discrimination is found to be related to the perception of religiosity or religion, religious or religion-specific taste-based discrimination might be measured rather than ethnic taste-based discrimination. However, if these other signals are of no importance for the measured ethnic discrimination, ethnic taste-based discrimination might be at play. Therefore, our research takes other “non-ethnic” signals in names into account and analyzes the underlying mechanisms of ethnic discrimination through the signals names carry.

## 3. Boundary work

When using names as signals in discrimination research, names are used by actors as a tool to define the in- and outgroup (Gerhards and Kämpfer, 2017). Which signal(s)—e.g. ethnic origin, socioeconomic status or religion/religiosity—is/are the trigger to discriminatory behavior, depends on which symbolic boundaries are the brightest in the studied context at that given moment, as the main feature of boundaries is that they are not solid but change across contexts throughout time (Conzen et al., 1992; Alba, 2005; Lamont et al., 2015). Bright boundaries are given social importance, are visible through emerging (cultural) differences between groups, are difficult to penetrate because of social closure and are therefore also durable (Lamont and Molnar, 2002; Alba, 2005; Wimmer, 2008). If groups have been able to differentiate themselves from others with great power and resources, they will make strategies like “boundary shifting” and “boundary crossing” more difficult. This means that the more inequality there is between groups, the more social closure and thus also the brighter the boundary (Wimmer, 2008).

Migration has been an important motive for the emergence of ethnic boundaries (Alba, 2005). This has led to the dialectical defining process of inclusion and exclusion. On the one hand, inclusion occurs as people with similar characteristics form a shared identity, which is acknowledged by the outgroup. On the other hand, exclusion from one group creates solidarity and thus the formation of a “counter-group” (Miles, 1991; Nagel, 1994; Zolberg and Woon, 1999; Fuller, 2003). Nevertheless, the symbolic location of the boundary dividing the groups can differ. A distinction can, for example, be made between immigrants and natives, or a more precise boundary can divide specific ethnic groups (Ponce, 2019). With the increasing Europeanisation and the emergence of a European identity, the ethnic boundary mainly distinguishes European from non-European citizens (Zolberg and Woon, 1999; Ponce, 2019). Consequently, the immigrant groups originating from other EU-member states are not perceived as “foreigners” as strongly as immigrant groups from non-EU countries (Jacobs et al., 2009). The latter contributes to the blurring of ethnic boundaries among ethnic groups of European origin.

Because of the color-blind ideology downplaying ethnic boundaries in (Western) Europe, other characteristics are emphasized to distinguish migrant-origin groups (Goldberg, 2006; Bail, 2008). The European identity is built upon a shared culture which is characterized by a set of norms and values as well as upon a shared history. The latter consists of (the emergence of) democracy, a “tradition of law and legal culture” and Christianity (Sztompka, 2004). It is especially this last element that lies at the basis of a bright boundary, differentiating historically (Judo-)- Christian but mostly secularizing Europe vs. Islam (Sztompka, 2004; Phalet et al., 2013; Ponce, 2019). Despite diversity within the Muslim population on different aspects (e.g., region of origin, ethnicity, phenotypical characteristics, or social class), they are categorized as one outgroup based on perceived threat to the European identity (Said, 1978; Zolberg and Woon, 1999).

This bright religious boundary is unlikely to blur in the near future, as the distance with the European (Judo-)Christian identity remains significant (Alba, 2005). This is visible through generally low levels of institutional accommodation of Islam (Phalet et al., 2013), which translates in for instance Muslim holidays and events not being officially recognized in spite of Christian ones. Additionally, if Muslims in Europe face inequality by being socioeconomically disadvantaged or by living in segregated areas, the boundary is maintained. Besides, when Muslims in Europe are confronted with discrimination and hostility, this contributes to the strengthening of the dialectical process defining the in- and out-group (Ponce, 2019). As boundaries are place and time dependent, certain boundaries are relevant in one place and irrelevant in another. Therefore, although in Western Europe the religious boundary is salient, this boundary can be more emphasized in certain countries than others based on the dominant political ideology (Kostoryano, 2004).

The human capital of the immigrant origin population might also be a relevant source of boundary creation (Bail, 2008; Neumann and Moy, 2018). Although on the social level and thus in the general public opinion, ethnic origin, culture or religion are salient topics, economic factors are found to be additionally relevant on the individual level (Sides and Citrin, 2007; Iyengar et al., 2013). Generally, natives in Europe appear to be in favor of skilled as opposed to unskilled immigrants, drawing a socioeconomic boundary (Bail, 2008; Facchini and Mayda, 2012; Iyengar et al., 2013). This is found to be driven both by (positive and negative) economic drivers and non-economic drivers (Facchini and Mayda, 2012). Positive economic drivers refer to skilled immigrants contributing to the welfare state, leading to an increased efficiency and innovation in production and conception and having a positive influence on the general price-levels of finished goods (Facchini and Mayda, 2012). As negative driver, the labor competition hypothesis predicts that native workers are more in favor of immigrants with other skills than their own. Consequently, if the native population is mostly highly educated, they might be less in favor of receiving highly educated immigrants. However, this hypothesis has been contested as some research found pro-skilled immigrant attitudes, regardless of the natives' skills (Hainmueller and Hiscox, 2007, 2010; Iyengar et al., 2013). Also, the fiscal burden hypothesis might act against the willingness to receive low-skilled immigrants. Following this hypothesis, immigrants can be perceived as an extra cost, either by increasing welfare state contributions or in terms of economic costs aimed at educating and providing healthcare for immigrants (Iyengar et al., 2013). Regarding non-economic drivers, skilled immigrants are thought to fit into the local culture more fluently and are perceived as less likely to be involved in illegal activities (Facchini and Mayda, 2012).

It follows that, depending on the context and time being studied, there may be several boundaries of interest that distinguish an “us” from a “them” group and may give rise to discrimination, among other things. These boundaries and their consequences can be extended to invisible signals in contexts where contact happens indirectly, like names in the first stage of the rental application process, and might explain—at least to some extent—measured differences in accessibility to rental housing.

## 4. Hypotheses

**Hypothesis 1:** Candidates with non-European names (here Moroccan names) are expected to face higher levels of discrimination than candidates with European names (here Polish names).

**Hypothesis 2:** The strength of ethnic signals (and thus brightness of ethnic boundaries) is expected to have an effect on the level of measured negative discrimination.

→ *Hypothesis 2A*: The perception of a name as European or not is expected to have a stronger effect on the measured negative discrimination than the precise ethnic origin perceived.

European–non-European boundary:

→ *Hypothesis 2B*: The perception of a Moroccan name as non-European is expected to increase the measured discrimination.
→ *Hypothesis 2C*: The perception of a Polish name as European is expected to decrease the measured discrimination.

Ethnic boundary based on precise ethnic origin:

→ *Hypothesis 2D*: The perception of a Moroccan name as Moroccan is expected to increase the measured discrimination.
→ *Hypothesis 2E*: The perception of a Polish name as Polish is expected to increase the measured discrimination.

**Hypothesis 3:** If we find that the perception of names as religious has an effect on the measured negative discrimination, we measure ethnic discrimination which arises through distastes toward religiosity. This is in line with taste-based discrimination, whereby tastes related to religiosity or the specific religion matter (religious and/or religion-specific taste-based discrimination).

→ If religiosity only has an effect on discrimination toward candidates with Moroccan names, we argue that we find religion-specific taste-based discrimination, whereby (dis)tastes are directed at Islam in spite of (Judo-)- Christianism.
→ If religiosity has an effect on discrimination toward both candidates with Moroccan and Polish names, we argue that we find religious rather than religion-specific taste-based discrimination.

**Hypothesis 4:** If we find that the perception of social class and educational level has an effect on the measured negative discrimination, we measure ethnic discrimination which arises through socioeconomic related stereotypes and prejudices. This is in line with statistical discrimination.

**Hypothesis 5:** If we find no effect of the perception of social class, educational level or religiosity in names on negative discrimination, then this is in line with taste-based discrimination, whereby tastes related to ethnic origin matter (ethnic taste-based discrimination).

## 5. Data and methodology

The data of this study consists of a combination of two different data collections: an online survey on the perception of names and correspondence tests on the rental housing market in Flanders. The respondents of the survey are not the same as the correspondence tested realtors and private landlords in order to avoid detection. Both data collections were performed between April and June 2021. The housing market in Flanders is dominated by home owners with outstanding mortgages, which were for long heavily subsidized by the government. Private renters account for a relatively small proportion of tenure types, and is especially popular among young people and lower income groups (Winters and Heylen, 2014). Almost no additional policy measures were taken during the Covid-19 pandemic to protect tenants or landlords (Dreesen and Heylen, 2023). The regional government only prevented forced evictions (e.g., through a temporary moratorium) and a tenant could request an extension of the rental agreement in the case of exceptional circumstances. Not surprisingly, the pandemic increased the existing affordability gap between homeowners and tenants in Flanders.

### 5.1. Survey on the perception of names

Firstly, we use data from an online survey conducted in April 2021 among a non-probability sample of 990 ethnic majority^1^ respondents living in Flanders in Belgium. The aim of the survey was to gain insight in the perception of names on signals of ethnic origin, gender, religiosity, social class and educational level. Therefore, we tested a selection of 180 combinations of first and last names. Every respondent had to rate 10 names; thus the 180 names were randomly divided over 18 sets. Within each set, the order of the names varied to avoid order-effects. The name combinations stem from official databases containing the most common male and female names between 2010 and 2019 and most common last names in 2020.^2^ We tested the perception of Belgian,^3^ Moroccan, Polish, Turkish, and Congolese names. A further distinction was made based on gender and mixed^4^ vs. homogenous names. For the present study, we solely take the perception of Belgian, Moroccan and Polish male and female homogenous names into account.^5^ These categories account for 60 out of the 180 names (20 names per ethnic group: 10 female and 10 male names).

For the perception of ethnic origin, the respondents could choose between seven answer categories: “Belgian”, “Another European origin”, “Non-European origin”, “Belgian + another European origin”, “Belgian + another non-European origin”, “Another European origin + non-European origin” and “Don't know”. If they opted for a category with “Another European origin” or “Non-European origin”, they were additionally asked to specify the ethnic origin in an open answer field. Based on these questions, we created congruence variables, which are dichotomous variables whereby “1” (as opposed to “0”) means that the respondent was successful in perceiving the intended ethnic signal (Gaddis, 2017). We created two types of congruency:

- The distinction between European and non-European origin: For Belgian names, we rated the option “Belgian” and “another European origin” as congruent. For Moroccan names, “Another non-European origin” and for Polish homogenous names “Another European origin” is seen as congruent.
- The congruence on the specific ethnic-national origin: Here, naming the correct country of origin (Morocco or Poland) in the open answer field was rated as congruent. As respondents were only asked to name a specific country of origin if they chose another answer category than “Belgian”, Belgian names have no congruence rate on the specific ethnic origin.

Afterwards, we calculated the congruence rate on the aggregated level of the name: the percentage respondents that correctly perceived the ethnic signals for each name, which is used in the current study as the congruence rate.

For the perception of religiosity, social class and educational level, the respondents were asked to rate their perception of each name on 7-point Likert scales with an 8^th^ option “Don't know”. These answers were recoded in three variables with three categories: (1) “Not religious”, (2) “Neutral” (including the option “Don't know”) and (3) Religious; (1) “Lower social class”, (2) “Middle social class” (including the option “Don't know”) and (3) “High social class”; (1) “Low educational level”, (2) “Middle educational level” (including the option “Don't know”) and (3) “High educational level”. For the current study, we calculated for each name the percentage respondents that perceive the name as religious, of a low social class and of a low educational level (aggregated level). Nevertheless, it might be that respondents would have perceived other signals or no signals in this regard if it was not asked for.

### 5.2. Correspondence tests

**Figure 1:**
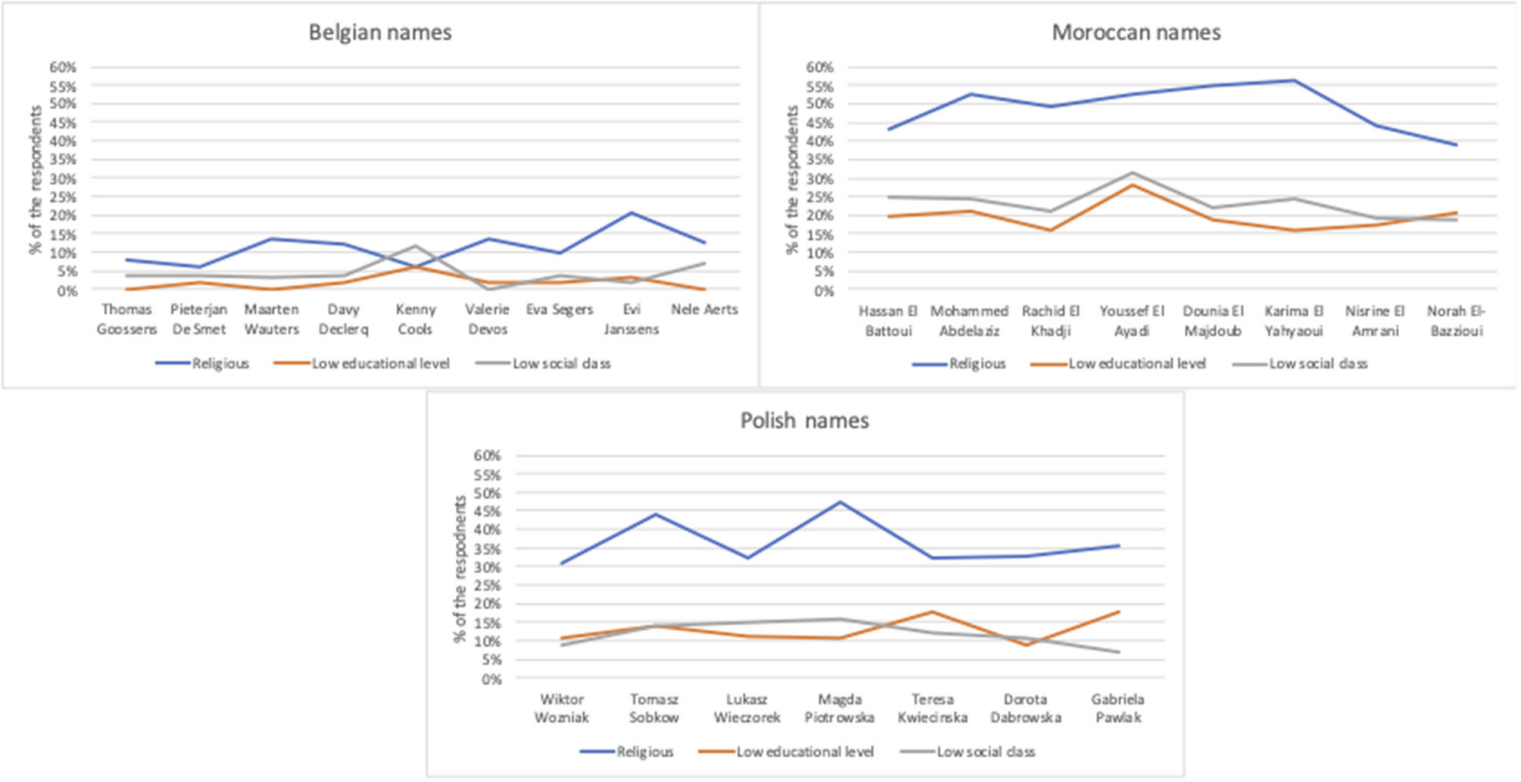
Individual name-ratings on religiosity, educational level and social class divided per ethnic group.

Between April and June 2021, we conducted 3.753 pairwise matched correspondence tests on the Flemish rental housing market. This method is, next to situation tests, a widely used method to measure rental discriminatory behavior in the field (Flage, 2018; Auspurg et al., 2019; Quillian et al., 2020). More specifically, we measure opportunity denying (Riach and Rich, 2002; Hogan and Berry, 2011) and thus whether or not the candidates are invited for a viewing. We test rental discrimination toward candidates with Moroccan names (non-European ethnic origin) and candidates with Polish names (European ethnic origin). In line with previous research, names function as the main signal of ethnic origin and gender (Flage, 2018). Both male and female names are taken into account. The names that are used in these correspondence tests are 24 out of the 60 Belgian, Polish and Moroccan names we thoroughly pre-tested in the online survey discussed above and are shown in Table 1. In total we tested 15 non-Belgian names in comparison to 9 Belgian names. We chose the names that appeared to have the clearest signal of ethnic origin in the survey. Additionally, we chose names that provide within-group variance in terms of the perception of religiosity, social class and educational level (Figure 1).

**Table 1:** The names from the online survey that are used in the correspondence tests.

	**Test profile**	**Control profile**
Moroccan man vs. Belgian man	Hassan El Battoui	Thomas Goossens
	Mohammed Abdelaziz	Pieterjan De Smet
	Rachid El Khadji	Maarten Wauters
	Youssef El Ayadi	Kenny Cools
Moroccan woman vs. Belgian woman	Dounia El Majdoub	Valerie Devos
	Karima El Yahyaoui	Eva Segers
	Nisrine El Amrani	Nele Aerts
	Norah El-Bazzioui	Evi Janssens
Polish man vs. Belgian man	Wiktor Wozniak	Maarten Wauters
	Tomasz Sobkow	Davy Declerq
	Lukasz Wieczorek	Kenny Cools
Polish woman vs. Belgian woman	Magda Piotrowska	Valerie Devos
	Teresa Kwiecinska	Eva Segers
	Dorota Dabrowska	Nele Aerts
	Gabriela Pawlak	Evi Janssens

During the tests, two (or three) rental candidates—one (or two) test profile(s) and one control profile—reply to the same rental advertisement on Immoweb and ask for a viewing.^6^ All candidates have similar profiles, except for their names. The names were visible in the e-mail address, the profile on Immoweb and the signature at the end of the message. We considered rental advertisements on Immoweb, because it is one of the largest rental websites in Belgium. Because most rental advertisements are proposed on multiple websites at the same time, we chose the most common website. To standardize the content of the messages, we used the standard issue message on Immoweb. By doing so, it is unlikely that a difference in treatment is due to a difference in the message. The order in which the fictive rental candidates replied to the rental advertisement was randomized. In order to avoid detection, we excluded dwellings with a rent above 1,500 euros as well as student residences and flats designed for elderly, because these specific market niches attract very specific target groups and often come along with more tailored selection procedures.

### 5.3. Method

The results are analyzed by considering the responses made by real estate agents and private landlords. Here, there are four possible response categories: (1) nor the test nor the control profile is invited for a viewing, (2) both the test and control profiles are invited for a viewing, (3) only the test profile is invited for a viewing and (4) only the control profile is invited for a viewing. In Table 2, we present the descriptive statistics for the four possible response categories. As is the case in most of the correspondence studies in Belgium, the most recurrent scenario is the one whereby neither the test nor the control profile is invited for a viewing (Verhaeghe and De Coninck, 2022; Ghekiere et al., 2023b). This varies from a total of 57.1% among realtors to 63.4% among private landlords. However, the second most frequent response is one where both the test- and control profiles are invited for a viewing (around 29.9% for realtors and 22.0% for private landlords). This is more the case in tests whereby the test profile is Polish (32.1 and 26.0% for realtors and private landlords respectively), than when the test profile is Moroccan (28.5 and 19.2% for realtors and private landlords respectively). Also, the situation in which only the control profile gets invited is more frequent as compared to the situation in which only the test person is invited for a viewing: 9.4% among realtors and 13.1% among private landlords as compared to 3.6% and 1.5% respectively.

**Table 2:** Descriptive statistics of the four possible response categories.

	**Realtors (*n =* 1,792)**			**Private landlords (*n =* 1,961)**		
	**Moroccan name**	**Polish name**	**Total**	**Moroccan name**	**Polish name**	**Total**
(1) Nor the test nor the control profile is invited for a viewing	57.40%	56.80%	57.10%	65.30%	60.7%	63.4%
(2) Both the test and control profiles are invited for a viewing	28.50%	32.10%	29.90%	19.20%	26.0%	22.0%
(3) Only the test profile is invited for a viewing	3.50%	3.80%	3.60%	1.20%	1.9%	1.5%
(4) Only the control profile is invited for a viewing	10.70%	7.40%	9.40%	14.20%	11.4%	13.1%

As we aim to focus on discrimination toward ethnic minority candidates and thus negative discrimination, we analyze category (4) compared to category (1), (2), and (3). We conduct binary logistic regression analysis, given that our dependent variable is a dichotomous variable. We conduct the analysis separately for realtors and private landlords, given their profoundly different selection behavior (Flage, 2018).

**Figure 2:**
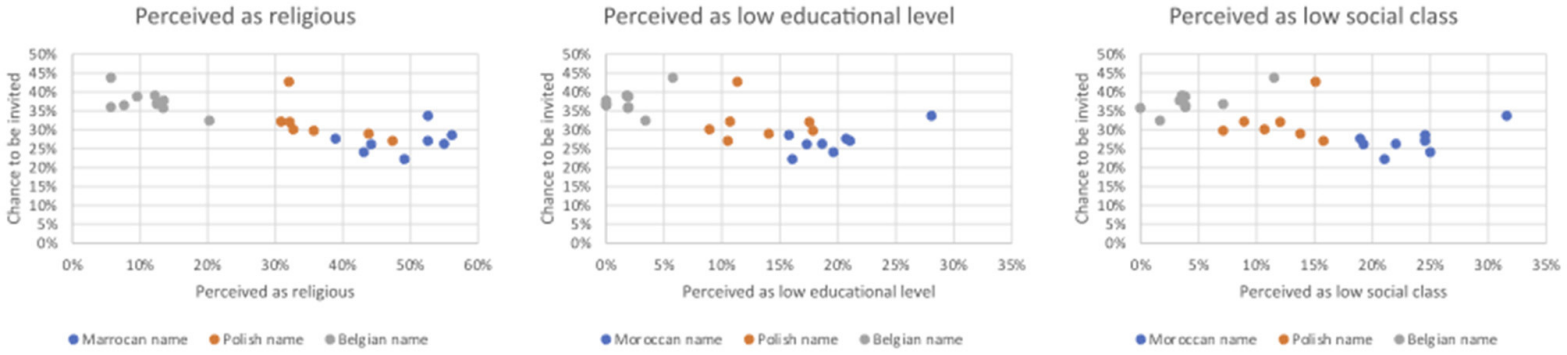
The invitation rates for each name plotted against religiosity, social class and educational level.

As seen in Figure 2, the ethnic origin of a name is correlated with the perception of a name as religious, low social class and low educational level. Non-Belgian names are rated as more religious, low social class and low educational level. This makes it difficult to disentangle ethnic origin effects from religiosity, class and educational effects. Therefore, we have to interpret our results with caution. To address this problem, we take solely the ethnic minority names into account in the binary logistic regression of discrimination. This leads to a somewhat better overlap in ratings between ethnic groups (Moroccan and Polish) and thus a better statistical comparability.

In addition, we analyze the odds to be invited for a viewing for both ethnic minority and majority groups as robustness check (see Appendix Tables 4, 5). We conduct multilevel analyses, because rental candidates are nested in rental advertisements. The variances on the advertisement-level are significant in the null model (*p* < 0.001). As invitation rates need to be compared to a reference category (here Belgian names) in order to infer discrimination, the ratings of Belgian names are included in these analysis. Both forms of analysis, however, come to similar results.

### 5.4. Variables

The dependent variable, when looking at the responses made by realtors and private landlords, is *discrimination*. This is a dichotomous variable whereby 1 stands for “negative discrimination” and 0 stands for “no negative discrimination”. We talk about discrimination when the control person was invited for a viewing, but not the test person.^7^

“*Moroccan name”* is a dichotomous variable whereby 0 “Belgian name” and 1 “Moroccan name”.

“*Polish name”* is a similar variable, with 0 “Belgian name” and 1 “Polish name”.

“*Gender”* is a dichotomous variable, with 0 “man” and 1 “women”.

“*Congruence European vs. Not-European origin”* and “*Congruence specific ethnic origin”* are continuous variables. They represent the percentage respondents that correctly perceived the ethnic signals in names on either the distinction between European and non-European names or the specific ethnic origin.

“*Religious'*, “*Low social class”* and “*Low educational level”* are continuous variables and represent the percentage respondents who perceive the names as religious, of a low social class or of a low educational level.

We take two control variables into account, namely the “*monthly rent”* and the “*number of bedrooms'*. Both are continuous variables.

Table 3 presents the descriptive statistics for the congruence rate variables and the perception of names as religious, of a low social class and low educational level. Respondents are better at categorizing Moroccan and Polish names as European or not than to define the specific ethnic origin. Besides, both types of congruence rates are higher for Polish as compared to Moroccan names. When looking at the means, the percentage respondents perceiving names as religious, of a low social class and low educational level is highest for Moroccan names and lowest for Belgian names.

**Table 3:** Descriptive statistics (in %).

		**Min**	**Max**	**Mean**	**SD**	**N_names_**
Congruence rate European vs. not-European origin	All	37.90	98.10	75.78	18.86	24
	Moroccan names	37.90	61.50	52.36	8.29	8
	Polish names	57.90	74.10	68.72	5.79	7
	Belgian names	80.40	98.10	92.72	4.57	9
Congruence rate specific ethnic origin	All	28.10	65.50	46.15	9.95	24
	Moroccan names	28.10	49.00	41.43	6.92	8
	Polish names	32.10	65.50	53.33	9.57	7
	Belgian names	-	-	-	-	9
Religious	All	5.80	56.10	27.22	16.97	24
	Moroccan names	39.00	56.10	47.69	5.57	8
	Polish names	30.90	47.40	35.56	5.77	7
	Belgian names	5.80	20.30	11.55	4.26	9
Low educational level	All	0.00	28.10	9.08	8.30	24
	Moroccan names	15.80	28.10	19.06	2.82	8
	Polish names	8.90	17.90	13.22	3.35	7
	Belgian names	0.00	5.80	1.41	1.64	9
Low social class	All	0.00	31.60	11.22	8.89	24
	Moroccan names	19.00	31.60	23.39	2.95	8
	Polish names	7.10	15.80	11.20	2.91	7
	Belgian names	0.00	11.50	3.88	2.52	9

## 6. Results

From the 3.753 correspondence tests, we found 10.7% discrimination against candidates with Moroccan names and 7.4% discrimination against candidates with Polish names among real estate agents. Among private landlords, these percentages are 14.2 and 11.4% respectively. Table 4 presents the binary logistic regression analysis on negative discrimination by ethnic origin. Notwithstanding the discrimination rates above, we do not find statistically significant differences in negative discrimination between rental candidates with Polish and Moroccan names. Rental candidates with a Moroccan name have 1.428 higher odds to be discriminated against by realtors compared to their Polish counterpart. However, this result is only borderline significant (*p* = 0.054). The result for private landlords is not significant. We therefore reject **hypothesis 1**, that expected to find more negative discrimination toward candidates with Moroccan as compared to Polish names.

**Table 4:** Binary logistic regression analysis on negative discrimination by ethnic origin.

	**Realtors (*n =* 1,792)**	**Private landlords (*n =* 1,961)**
	**OR (SE)**	**OR (SE)**
Constant	0.091 (0.322)^***^	0.144 (0.279)^***^
Moroccan name (ref. Polish name)	1.428 (0.185)^†^	1.204 (0.146)
Gender (ref. man)	0.700 (0.182)^†^	0.893 (0.143)
Monthly rent	1.001 (0.001)	1.001 (0.001)
Number of bedrooms	0.980 (0.123)	0.992 (0.045)
AIC	1,043.5	1,433.7

OR, Odds ratios; SE, Standard errors; ref., Reference category. ^***^p < 0.001; ^**^p < 0.01; ^*^p < 0.05;^†^*p* < 0.01.

Tables 5, 6 present the binary logistic regression analysis on discrimination by realtors and private landlords respectively. First, we look at whether the level of discrimination varies depending on how strong the signals of ethnic origin are. The latter was operationalized by looking at the congruence rate, which refers to the percentage respondents that successfully perceived the ethnic signals in names. We conducted the analysis separately for the congruent perception of names as being European or not and for the congruent perception of the specific ethnic origin. Besides, we consider the perception of a name as religious, of a low social class and educational level. We also present two full models (one including the congruency for European vs. non-European and one for the congruency on specific ethnic origin). Social class is excluded from these full models, because of its correlation with religiosity and educational level (r > 0.6).

**Table 5:** Binary logistic regression on negative discrimination by real estate agents (*n* = 1,792).

	**Congruence on European vs. non-European origin**	**Congruence on specific ethnic origin**	**Religiosity**	**Social class**	**Educational level**	**Full model 1—Congruence European vs. non-European origin**	**Full model 2—Congruence specific ethnic origin**
	**Model 1**	**Model 2**	**Model 3**	**Model 4**	**Model 5**	**Model 6**	**Model 7**
	**OR (SE)**	**OR (SE)**	**OR (SE)**	**OR (SE)**	**OR (SE)**	**OR (SE)**	**OR (SE)**
Constant	0.123 (0.291)^***^	0.120 (0.28)^***^	0.041 (0.562)^***^	0.061 (0.423)^***^	0.051 (0.485)^***^	0.035 (0.608)^***^	0.033 (0.610)^***^
Gender (ref. man)	0.620 (0.196)^*^	0.648 (0.182)^*^	0.659 (0.180)^*^	0.745 (0.189)	0.694 (0.182)^*^	0.668 (0.199)^*^	0.700 (0.184)^*^
Monthly rent	1.001 (0.001)	1.001 (0.001)	1.001 (0.001)	1.001 (0.001)	1.001 (0.001)	1.001 (0.001)	1.001 (0.001)
Number of bedrooms	0.966 (0.124)	0.966 (0.125)	0.968 (0.124)	0.978 (0.124)	0.972 (0.123)	0.964 (0.124)	0.968 (0.125)
Congruence rate	0.991 (0.013)	0.995 (0.012)	-	-	-	0.995 (0.013)	1.000 (0.012)
Religious			1.024 (0.011)^*^	-	-	1.015 (0.013)	1.016 (0.013)
Low social class				1.031 (0.014)^*^	-	-	-
Low educational level					1.045 (0.020)^*^	1.029 (0.023)	1.029 (0.023)
AIC	1,046.7	1,047.1	1,042.3	1,042.4	1,042.4	1,044.6	1,044.7

OR, Odds ratios; SE, Standard errors; ref., Reference category. ^***^p < 0.001; ^**^p < 0.01; ^*^p < 0.05;^†^*p* < 0.01.

**Table 6:** Binary logistic regression on negative discrimination by private landlords (*n* = 1,961).

	**Congruence on European vs. non-European origin**	**Congruence on specific ethnic origin**	**Religiosity**	**Social class**	**Educational level**	**Full model 1—Congruence European vs. non-European origin**	**Full model 2—Congruence specific ethnic origin**
	**Model 1**	**Model 2**	**Model 3**	**Model 4**	**Model 5**	**Model 6**	**Model 7**
	**OR (SE)**	**OR (SE)**	**OR (SE)**	**OR (SE)**	**OR (SE)**	**OR (SE)**	**OR (SE)**
Constant	0.148 (0.263)^***^	0.160 (0.260)^***^	0.119 (0.449)^***^	0.112 (0.346)^***^	0.121 (0.412)^***^	0.082 (0.497)^***^	0.093 (0.494)^***^
Gender (ref. man)	1.016 (0.150)	0.882 (0.141)	0.869 (0.141)	0.930 (0.148)	0.874 (0.142)	1.037 (0.151)	0.891 (0.142)
Monthly rent	1.001 (0.001)	1.001 (0.001)	1.001 (0.001)	1.000 (0.001)	1.000 (0.001)	1.001 (0.001)	1.001 (0.001)
Number of bedrooms	0.992 (0.042)	0.993 (0.042)	0.992 (0.046)	0.991 (0.045)	0.991 (0.046)	0.992 (0.042)	0.993 (0.042)
Congruence rate	1.034 (0.011)^**^	1.015 (0.009)^†^	-	-	-	1.035 (0.011)^**^	1.017 (0.009)^†^
Religious			1.008 (0.009)	-	-	1.009 (0.010)	1.008 (0.009)
Low social class				1.019 (0.011)^†^	-	-	-
Low educational level					1.017 (0.017)	1.011 (0.020)	1.012 (0.019)
AIC	1,425	1,432.4	1,434.6	1,432.4	1,434.4	1,427	1,434.7

OR, Odds ratios; SE, Standard errors; ref., Reference category. ^***^p < 0.001; ^**^p < 0.01; ^*^p < 0.05;^†^*p* < 0.01.

**Figure 3:**
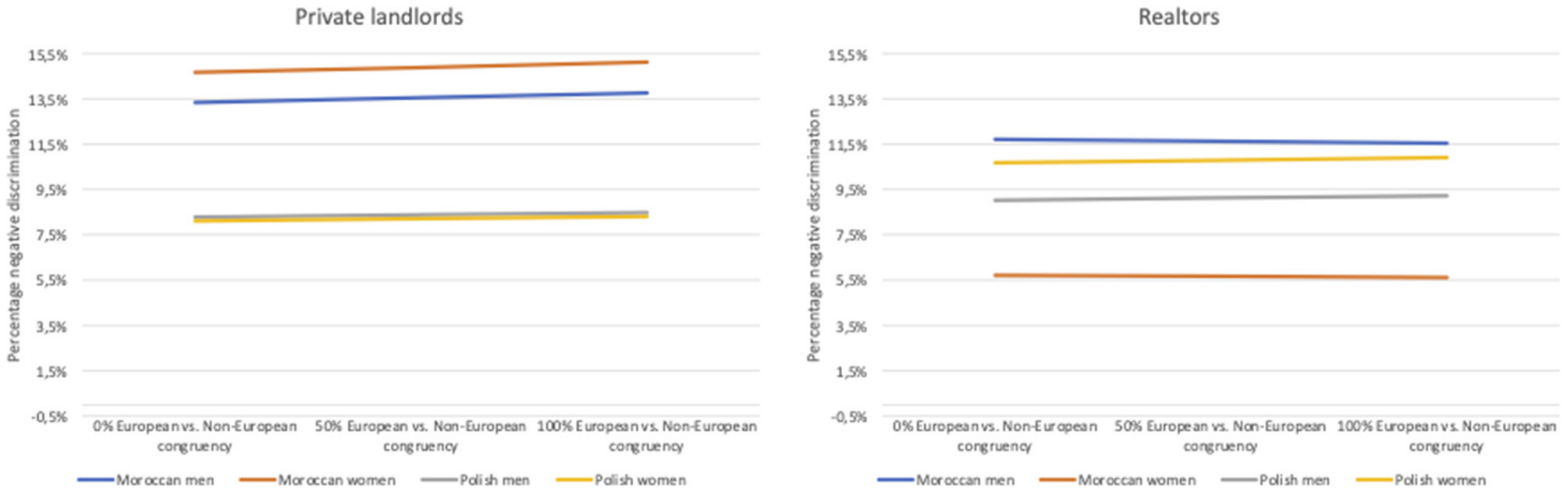
The effect of the correct perception of a name as European or not on the percentage negative discrimination, controlled for the number of bedrooms.

Firstly, although the congruent perception of a name as European or not has no influence on discrimination by realtors, it does for private landlords. Per one percent increase in the number of respondents that successfully categorized names as European or not, the chance to be discriminated by private landlords increases with 50.8%^8^ in Flanders. When analyzing the Polish and Moroccan names separately in Figure 3 (based on Appendix Tables 1, 2), this finding concerns only candidates with Moroccan names: although the effect is modest, the more Moroccan names are perceived as non-European, the higher the rental discrimination by private landlords toward these candidates. This comes also to the fore in Appendix Table 3: when considering only names with a congruency rate of 50% or more on the perception of a name as European or not, candidates with a Moroccan name face significantly more discrimination than candidates with Polish names. The congruent perception of a name as European or not has no effect on the measured discrimination toward candidates with Polish names.

The congruence rate on the perception of the specific ethnic origin of a name (models 2) has no influence on the level of discrimination by realtors. Regarding private landlords, the congruent perception of the specific ethnic origin of a name is borderline significant. However, if we split the results according to rental candidates' names (Appendix Table 1), neither effect is significant. Consequently, it is rather the European ethnic boundary that appears to be most influential and this for non-European names. This findings are also confirmed in the full models (Table 6, models 6 and 7) and support **hypothesis 2A and 2B**: the clearer the signals names carry as being of non-European origin—and thus the clearer the ethnic European boundary, the higher the level of rental discrimination by private landlords.

Although the origin of a name origin is of importance for discrimination by private landlords, gender discrimination is at play among realtors (Table 5). Women are less likely to be discriminated against as compared to their male counterpart. When considering the intersection with ethnic origin in Appendix Table 2, it appears that it is especially Moroccan women that are less discriminated against as compared to Moroccan men. This gender difference is not found among candidates with Polish names.

Secondly, perceiving a name as religious increases the odds to be discriminated against by 1.024 by realtors for both Moroccan and Polish names. The perception of religiosity in names does not appear to be influential for negative discrimination by private landlords. Thus, we find support for **hypothesis 3:** Real estate agents discriminate against ethnic minorities because of distastes toward religiosity, which is in line with taste-based discrimination in a color-blind ideology, whereby distastes are directed at perceived religiosity. We do not find this result for private landlords.

Thirdly, negative discrimination by realtors seems to also be based on the perception of a name as of a low social class and educational level. The higher the percentage respondents that perceive a name as of a low social class and educational level, the higher the ethnic discrimination by realtors. This supports **hypothesis 4** and aligns with statistical discrimination, whereby stereotypes and prejudices about a candidate's ability to pay rent and take care of the dwelling matter. The perception of a name as of a low social class has a borderline significant effect on negative discrimination by private landlords and this effect is rather small. Consequently, the results for private landlords indicate ethnic taste-based discrimination, as neither the perception of religiosity, social class or educational level have a significant effect on the measured discrimination. This supports **hypothesis 5**.

### 6.1. Robustness check

In Appendix Tables 4, 5, we perform multilevel logistic regression analysis on the odds to be invited for a viewing by realtors and private landlords respectively. Candidates with Moroccan and Polish names have significantly lower odds to be invited for a viewing than their Belgian counterparts. Regarding realtors, the perception of a name as religious mediates the effect of ethnic origin on the odds to be invited. When taking up both ethnic origin and the perception of religiosity in the same model, only the perception of a name as religious significantly decreases the odds to be invited for a viewing by realtors. Consequently, real estate agents discriminate against ethnic minorities because of distastes toward religiosity, which is in line with taste-based discrimination. As this is the case for both candidates with Moroccan and Polish names, and thus candidates with a migration background from countries with Muslim and Christian roots respectively, we find religious and not religion-specific taste-based discrimination. We do not find this result for private landlords. For the latter, nor the perception of religiosity, nor the perception of a name as of a low social class and educational level mediates the effect of ethnic origin. Consequently, private landlords discriminate toward ethnic minorities because of distastes toward certain minority groups, which is in line with ethnic taste-based discrimination.

## 7. Discussion and conclusion

Ethnic discrimination on the rental housing market is a well-documented fact that knows no spatial or periodical boundaries (Flage, 2018; Auspurg et al., 2019; Gaddis, 2019c). Often, correspondence testing is used to objectively capture discriminatory behavior in which it uses names as signals of ethnic origin (Bertrand and Mullainathan, 2004). Although this methodology is well-suited to describe the level and patterns of discrimination, it is—when used on its own—less suited to explain the underlying mechanisms (Gaddis, 2019c). This study aims to exploit the potential of signals carried by names in order to analyze how the measured rental discrimination can be explained. We, therefore, relate a thorough pretest of the subjective perception of names in terms of ethnic origin, religiosity, social class and educational level to objectively measured adverse treatment in the first phase of the rental process. The data consists of a combination of survey-data on the perception of Moroccan, Polish and Belgian names and 3.753 correspondence tests using these names on the rental housing market in Flanders, the Dutch speaking part of Belgium.

Based on our research, we find no significant indications that candidates with Moroccan names are confronted with higher levels of rental discrimination as compared to candidates with Polish names. However, when considering only names with clear signals as being European or not, Moroccan names face higher discrimination by private landlords than Polish names, but not by realtors. This is partly a variation on other research conducted in other European countries, such as Italy (Baldini and Federici, 2011), Sweden (Ahmed and Hammarstedt, 2008), Norway (Andersson et al., 2012), Belgium (Heylen and Van den Broeck, 2016; Verhaeghe and Ghekiere, 2021), Spain (Bosch et al., 2010), France (Le Gallo et al., 2019), and Germany (Auspurg et al., 2017) that systematically found higher discrimination rates against candidates with Arab/Muslim names as compared to other ethnic minority candidates.

Although both realtors and private landlords discriminate against candidates with Moroccan and Polish names, the underlying drivers appear to be different for both types of actors. Realtors are found to discriminate because of “(dis)tastes” toward the perceived religiosity of rental candidates, which is in line with taste-based discrimination. Because we find that perceiving a name as more religious increases discrimination against both candidates with Moroccan and Polish names, it might imply that it is religious rather than religion-specific taste-based discrimination that is at play. Moroccan names often have Islamic roots, whereas Polish names rather have a Christian influence (Khosravi, 2012; Cerchiaro, 2017). If religion-specific taste-based discrimination would be the main driver, the perception of a name as religious should only increase discrimination against candidates whose names have another religious background (here Islam) than that of the studied context (here Judo-Christianism in Belgium) (Sztompka, 2004; Phalet et al., 2013; Ponce, 2019). This finding can be described as “neo-racism” and is the product of color-blind ideology. In the absence or denial of strong ethnic boundaries, other but related characteristics than ethnic origin are used to differentiate the ethnic groups, in this case the religious boundary (Bonilla-Silva, 2003; Bail, 2008). Consequently, ethnic discrimination persists, as again shown in this study.

The finding that perceived religiosity increases discrimination by realtors against all minority candidates might contribute to the explanation of absence of significant differences in discrimination between Polish and Moroccan names. The increasing European identity gives strong importance to the distinction between historically (Judo-)Christian but secularizing Europe on the one hand and other religions, specifically Islam, on the other (Zolberg and Woon, 1999; Ponce, 2019). Consequently, an important characteristic of the European identity is secularizing Christianism (Zolberg and Woon, 1999; Phalet et al., 2013). The latter element might lay at the basis of a boundary dividing European countries based on how strong the secularizing process is pronounced. This boundary might explain why certain candidates with a European, but more Christian, migration background still face discrimination by realtors and why this discrimination increases with the perceived signals of religiosity. Future research could analyze this further by comparing the level of discrimination between rental candidates originating from more and less secularized European countries.

Besides, the stronger Moroccan and Polish names are perceived as “low social class” and “low educational level”, the higher the measured discrimination by realtors. This points toward statistical discrimination: rental candidates with Moroccan and Polish names are discriminated against because of existing stereotypes and prejudices about their ability to pay rent and take care of a rental dwelling (Carpusor and Loges, 2006). The latter result is in line with previous research, which found that giving more positive information about a candidate's professional or personal characteristics relates to lower levels of discrimination toward ethnic minority candidates (Baldini and Federici, 2011; Andersson et al., 2012). This was explained by the increased visible solvability of the rental candidate, which should lead to a lower perceived risk for the realtor.

When analyzing rental discrimination by private landlords, we find indications for classic ethnic taste-based discrimination. Private landlords are found to mainly discriminate against ethnic minority candidates because of personal “(dis)tastes” toward certain ethnic minority groups. The increased Europeanization and the emergence of a European identity created a European ethnic boundary differentiating European-migrants from non-European migrants (Ponce, 2019). Consequently, ethnic minorities originating from another European country, like candidates with Polish names, are perceived as less “foreign” as compared to ethnic minorities originating from a non-European country, like candidates with Moroccan names (Jacobs et al., 2009). It is especially this European ethnic boundary, and thus the perception of a name as European or not, that triggers discrimination by private landlords. The stronger the signal of a Moroccan name as non-European, the higher the levels of discrimination.

Although the origin of a name directly relates to discriminatory behavior among private landlords, gender discrimination is measured among realtors. Female rental candidates are less likely to be discriminated against as compared to male candidates. However, an intersection is at play with having a Moroccan name: the gender advantage found for women only holds among the candidates with Moroccan names, and thus not among the candidates with Polish names.

These results have implications for methodologies like correspondence testing that generally use names as the only signal. First, names carry rich and multiple signals. When thoroughly testing these signals, they can be used as indications as to what type of discrimination (e.g., statistical or taste-based) is at play. Second, if researchers have in-depth knowledge about the signals conveyed by the names used, these signals can be adjusted or additional signals can be added in a much more accurate way for deeper analysis. By doing so, more precise measures of the proportion of statistical and taste-based discrimination might be uncovered.

Moreover, this study has also a few policy implications. The current policies to combat rental discrimination in Belgium range from voluntary diversity training courses for realtors and landlords to applying correspondence testing as a policy tool. Although the long-term effects of voluntary trainings are still contested (Paluck and Green, 2009), a recent study among students in real estate showed that diversity trainings could be effective to lower statistical discrimination (Ghekiere et al., 2023a). The challenge for future trainings will be to tailor its content is such a way that it especially debunk the religious taste-based discrimination mechanism among realtors and the ethnic taste-based discrimination among private landlords. In addition, several cities in Belgium are currently experimenting with correspondence testing to raise awareness about discrimination and in a few cases even to sue discriminatory realtors [for an overview see Verhaeghe (2023)]. To be effective, these tests could consciously use the ethnic, religious and socioeconomic signals of names in a smart way, because this study shows that the strength of these signals relates to different levels of discrimination. Notwithstanding their individual strength, the use of both compliance (e.g., diversity trainings) and deterrence (e.g., correspondence testing) instruments are found to be most effective (Verstraete et al., 2017).

The results of this study must be read within the confines of a few limitations. Firstly, in this study we analyze signals in names separately in their relation to measured discrimination, whereas in real life the combination of different signals might trigger discrimination. In line with the latter, also other elements than signals in names might explain discriminatory behavior (f.e. the social and economic composition of the neighborhood of the dwelling, personal characteristics of the realtor or landlord,..). Secondly, the respondents of the survey in which the perception of names were tested are not the tested realtors and landlords whose behavior was investigated during the correspondence tests. Using the same sample in both data collections would increase the likelihood of detection. Realtors and landlords might have, however, different ethnic perceptions of names than the general public. Thirdly, because we conducted correspondence testing, we only measure discrimination in the first phase of the rental process. Discrimination, however, can occur throughout the whole rental process. Fourthly, in this study we measure opportunity denying. This means that the real proportion of discrimination is underestimated, given the parallel practice of opportunity diminishing (Riach and Rich, 2002; Hogan and Berry, 2011). The latter refers to being shown or told about fewer rental dwellings, being imposed additional rental conditions or being given less information. Also, by solely considering rental advertisements on an official rental website, we have no insight in informal rental processes. Finally, although we have information about how religious or not respondents perceive a name, we lack information about what specific religious denomination they would ascribe the name to. Nonetheless these caveats, this study highlights the complexity of signals that names bear, which can have an explanatory potential for the underlying mechanisms driving discrimination.

## Data availability statement

The data supporting the conclusions of this article are available from the researchers on reasonable request.

## Ethics statement

Ethical approval has been granted within the EdisTools Project to conduct questionnaires among human participants in which participation was voluntary and after informed consent. This ethical approval for conducting the survey has been granted by the ethical commission of the Political and Social Sciences of Ghent University. The data were processed and analyzed anonymously. For the correspondence tests, ethical approval was granted by the Ethical Committee for Social and Behavioral Sciences of the Vrije Universiteit Brussel (ECHW number 289). The Ethics Committee waived the requirement of written informed consent for participation.

## Author contributions

BM created the conception of the study, organized the database, performed the statistical analysis, and wrote the first draft of the manuscript. Both authors contributed to manuscript revision, read, and approved the submitted version.

## Footnotes

^1^“Ethnic majority” is operationalized as having the Belgian nationality and the person's parents are born in Belgium.

^2^This data was accessed *via* https://statbel.fgov.be/fr/themes/population.

^3^By choosing names that are common in Belgium, this provides strong indications that those names are Belgian. Although traditionally there is a difference in typically Flemish and Walloon (last) names, with the important internal migration in Belgium (Martiniello and Rea, 2012), names that are traditionally more Flemish are also strongly present in Wallonia and vice versa. Therefore, both Flemish and Walloon names are likely to be perceived by a Belgian sample as Belgian names. This was indeed the case in our survey, as 79.5% of the respondents perceived the names we used to signal a Belgian origin as such (available upon request).

^4^“Mixed names” is operationalized as having a Belgian first name and a non-Belgian last name.

^5^We opt for Moroccan and Polish names in comparison to Belgian names, because we want to compare a European to a non-European ethnic origin. The choice for Moroccan over Turkish and Congolese names, is to be in line with previous research (e.g., Ahmed and Hammarstedt, 2008; Bosch et al., 2010; Baldini and Federici, 2011; Andersson et al., 2012; Heylen and Van den Broeck, 2016; Auspurg et al., 2017; Le Gallo et al., 2019; Verhaeghe and Ghekiere, 2021) and to test whether we find similar results when using thoroughly pre-tested names.

^6^The use of duo or triad tests has no effect on the measured level of discrimination (Ghekiere et al., 2022).

^7^For the robustness check, which focuses on individual responses by means of multilevel analysis, the dependent variable is *the odds to be invited for a viewing*, with 1 “invitation” and 0 “no invitation”. We talk about an invitation when this is explicitly mentioned in the reaction of the realtor or landlord: suggestion for a group viewing, asks to send some possibilities to come by, asks to call the office to arrange the viewing.

^8^From odds ratios to chance: OR/1+OR (1.034/2.034) = 0.5084.
