# Supplementary material for: Different names, different discrimination? How perceptions of names can explain rental discrimination
Source: Front Sociol. 2023 Mar 2;8:1125384. doi: 10.3389/fsoc.2023.1125384 (PMC10018195; doi:10.3389/fsoc.2023.1125384)
Supplement: Supplementary file 1 [file Data_Sheet_1.docx]

1. Appendix

***Table I. Binary logistic regression on discrimination towards Moroccan and Polish rental candidates by private landlords, controlled for the congruence rates***

|  |  |  |  |  |
| --- | --- | --- | --- | --- |
|  | **Moroccan name (n=1165)** | | **Polish name (n=796)** | |
|  | ***Congruence on European vs. Non-European origin*** | ***Congruence on specific ethnic origin*** | ***Congruence on European vs. Non-European origin*** | ***Congruence on specific ethnic origin*** |
|  | **Model 1A** | **Model 2A** | **Model 1B** | **Model 2B** |
|  | *OR (SE)* | *OR (SE)* | *OR (SE)* | *OR (SE)* |
| Constant | 0.154 (0.323)*** | 0.181 (0.314)*** | 0.129 (0.451)*** | 0.120 (0.460)*** |
| Gender (ref. man) | 1.112 (0.201) | 0.873 (0.184) | 0.979 (0.236) | 0.970 (0.234) |
| Monthly rent | 0.999 (0.001) | 0.999 (0.001) | 1.001 (0.001) | 1.001 (0.001) |
| Number of bedrooms | 0.999 (0.035) | 1.001 (0.035) | 0.836 (0.179) | 0.838 (0.179) |
| Congruence rate | 1.037 (0.012)** | 1.016 (0.012) | 1.026 (0.021) | 1.016 (0.013) |
| AIC | 889.45 | 896.73 | 542.54 | 542.55 |
| OR = Odds ratios; SE = Standard errors; ref. = Reference category | | | | |
| ***p<0.001;**p<0.01;*p<0.05; †p<0.1 | | | | |
|  |  |  |  |  |

***Table II. Binary logistic regression on discrimination towards Moroccan and Polish rental candidates by realtors, controlled for the congruence rates***

|  |  |  |  |  |
| --- | --- | --- | --- | --- |
|  | **Moroccan name (n=1100)** | | **Polish name (n=692)** | |
|  | ***Congruence on European vs. Non-European origin*** | ***Congruence on specific ethnic origin*** | ***Congruence on European vs. Non-European origin*** | ***Congruence on specific ethnic origin*** |
|  | **Model 1A** | **Model 2A** | **Model 1B** | **Model 2B** |
| Constant | 0.126 (0.338)*** | 0.114 (0.330)*** | 0.131 (0.585)*** | 0.123 (0.591)*** |
| Gender (ref. man) | 0.457 (0.269)*** | 0.532 (0.242)*** | 1.205 (0.317) | 1.235 (0.320) |
| Monthly rent | 1.001 (0.001) | 1.001 (0.001) | 0.999 (0.001) | 0.999 (0.001) |
| Number of bedrooms | 1.028 (0.143) | 1.030 (0.144) | 0.870 (0.243) | 0.882 (0.245) |
| Congruence rate | 0.982 (0.014) | 0.991 (0.014) | 1.024 (0.029) | 1.017 (0.018) |
| AIC | 698.58 | 699.79 | 346.59 | 346.41 |
| OR = Odds ratios; SE = Standard errors; ref. = Reference category | | | | |
| ***p<0.001;**p<0.01;*p<0.05; †p<0.1 | | | | |

***Table III. Binary logistic regression analysis on negative discrimination by ethnic origin – extended table***

|  |  |  |  |  |
| --- | --- | --- | --- | --- |
|  | **All names** | | **>= 50% congruence European vs. Non-European origin** | |
|  | **Realtors (n=1792)** | **Private landlords (n=1961)** | **Realtors (n=1353)** | **Private landlords (n=1522)** |
|  | *OR (SE)* | *OR (SE)* | *OR (SE)* | *OR (SE)* |
| Constant | 0.091 (0.322)*** | 0.144 (0.279)*** | 0.085 (0.386)*** | 0.130 (0.315)*** |
| Moroccan name (ref. Polish name) | 1.428 (0.185)† | 1.204 (0.146) | 1.294 (0.210) | 1.477 (0.157)* |
| Gender (ref. man) | 0.700 (0.182)† | 0.893 (0.143) | 0.656 (0.226) | 0.926 (0.158) |
| Monthly rent | 1.001 (0.001) | 1.001 (0.001) | 1.001 (0.001) | 1.001 (0.001) |
| Number of bedrooms | 0.980 (0.123) | 0.992 (0.045) | 0.893 (0.160) | 0.993 (0.082) |
| AIC | 1043.5 | 1433.7 | 739.1 | 1157.4 |
| OR = Odds ratios; SE = Standard errors; ref. = Reference category | | |  |  |
| ***p<0.001;**p<0.01;*p<0.05; †p<0.01 | | |  |  |
|  |  |  |  |  |

***Robustness check***

***Table IV. Multilevel logistic regression analysis on the odds to be invited for a viewing by real estate agents (n=3584)***

|  |  |  |  |  |  |  |  |  |  |
| --- | --- | --- | --- | --- | --- | --- | --- | --- | --- |
|  |  | **Congruence European vs. Non-European origin** | | **Religiosity** | | **Social class** | | **Educational level** | |
|  | **Model 1** | **Model 2A** | **Model 3A** | **Model 2B** | **Model 3B** | **Model 2C** | **Model 3C** | **Model 2D** | **Model 3D** |
|  | *OR (SE)* | *OR (SE)* | *OR (SE)* | *OR (SE)* | *OR (SE)* | *OR (SE)* | *OR (SE)* | *OR (SE)* | *OR (SE)* |
| Constant | 0.106 (0.505)*** | 0.080 (0.002)*** | 0.111 (0.504)*** | 0.113 (0.505)*** | 0.137 (0.523)*** | 0.098 (0.496)*** | 0.112 (0.515)*** | 0.098 (0.497)*** | 0.104 (0.510)*** |
| Moroccan name (ref. Belgian name) | 0.512 (0.139)*** | - | 0.519 (0.139)*** | - | 1.434 (0.512) | - | 0.606 (0.551) | - | 0.434 (0.556) |
| Polish name (ref. Belgian name) | 0.617 (0.166)** | - | 0.630 (0.166)*** | - | 1.188 (0.355) | - | 0.655 (0.263) | - | 0.555 (0.384) |
| Gender (ref. man) | 0.746 (0.163) | - | 0.719 (0.163)* | - | 0.813 (0.168) | - | 0.735 (0.179) | - | 0.743 (0.163) |
| Congruence rate | - | 0.989 (0.002)*** | 0.992 (0.011) | - | - | - | - | - | - |
| Religious | - | - | - | 0.981 (0.003)*** | 0.972 (0.014)* | - | - | - | - |
| Low social class | - | - | - | - | - | 0.968 (0.007)*** | 0.992 (0.027) | - | - |
| Low educational level | - | - | - | - | - | - | - | 0.965 (0.007)*** | 1.001 (0.030) |
| Monthly rent | 1.001 (0.001) | 1.001 (0.001)*** | 1.001 (0.001) | 1.001 (0.001) | 1.001 (0.001) | 1.001 (0.001) | 1.001 (0.001) | 1.001 (0.001) | 1.001 (0.001) |
| Number of bedrooms | 1.116 (0.197) | 1.002 (0.002)*** | 1.144 (0.198) | 1.121 (0.198) | 1.129 (0.199) | 1.107 (0.195) | 1.066 (0.200) | 1.118 (0.196) | 1.114 (0.197) |
| AIC | 3681.3 | 3709.9 | 3682.7 | 3675.6 | 3678.9 | 3685.2 | 3683.3 | 3682.7 | 3683.2 |
| OR = Odds ratios; SE = Standard errors; ref. = Reference category | | | | | | | | | |
| ***p<0.001;**p<0.01;*p<0.05; †p<0.10 | | | | | | | | | |
|  |  |  |  |  |  |  |  |  |  |

***Table V. Multilevel logistic regression analysis on the odds to be invited for a viewing by private landlords (n=3922)***

|  |  |  |  |  |  |  |  |  |  |
| --- | --- | --- | --- | --- | --- | --- | --- | --- | --- |
|  |  | **Congruence European vs. Non-European origin** | | **Religiosity** | | **Social class** | | **Educational level** | |
|  | **Model 1** | **Model 2A** | **Model 3A** | **Model 2B** | **Model 3B** | **Model 2C** | **Model 3C** | **Model 2D** | **Model 3D** |
|  | *OR (SE)* | *OR (SE)* | *OR (SE)* | *OR (SE)* | *OR (SE)* | *OR (SE)* | *OR (SE)* | *OR (SE)* | *OR (SE)* |
| Constant | 0.002 (0.683)*** | 0.048 (0.468)*** | 0.002 (0.688)*** | 0.040 (1.053)** | 0.002 (0.710)*** | 0.050 (0.648)*** | 0.002 (0.698)*** | 0.002 (0.655)*** | 0.002 (0.687)*** |
| Moroccan name (ref. Belgian name) | 0.069 (0.238)*** | - | 0.065 (0.242)*** | - | 0.143 (0.741)** | - | 0.094 (0.759)** | - | 0.112 (0.685)** |
| Polish name (ref. Belgian name) | 0.203 (0.227)*** | - | 0.211 (0.227)*** | - | 0.318 (0.491)* | - | 0.228 (0.348)*** | - | 0.283 (0.494)* |
| Gender (ref. man) | 0.223 (0.204)*** | - | 0.246 (0.205)*** | - | 0.233 (0.207)*** | - | 0.218 (0.212)*** | - | 0.226 (0.204)*** |
| Congruence | - | 0.976 (0.010)* | 0.955 (0.016)** | - | - | - | - | - | - |
| Religious | - | - | - | 0.956 (0.006)*** | 0.981 (0.019) | - | - | - | - |
| Low social class | - | - | - | - | - | 0.926 (0.008)*** | 0.984 (0.038) | - | - |
| Low educational level | - | - | - | - | - | - | - | 0.877 (0.011)*** | 0.973 (0.037) |
| Monthly rent | 1.002 (0.001)* | 1.003 (0.001)*** | 1.002 (0.001)* | 1.004 (0.001)*** | 1.002 (0.001)* | 1.003 (0.001)*** | 1.002 (0.001)* | 1.002 (0.001)* | 1.002 (0.001)* |
| Number of bedrooms | 0.453 (0.283)** | 0.432 (0.172)*** | 0.435 (0.285)** | 0.353 (0.227)*** | 0.450 (0.283)** | 0.379 (0.203)*** | 0.454 (0.283)** | 0.512 (0.265)* | 0.456 (0.283)** |
| AIC | 3611.5 | 3825.0 | 3604.7 | 3683.8 | 3612.4 | 3712.1 | 3613.3 | 3673.0 | 3612.9 |
| OR = Odds ratios; SE = Standard errors; ref. = Reference category | | | | | | | | | |
| ***p<0.001;**p<0.01;*p<0.05; †p<0.10 | | | | | | | | | |
|  |  |  |  |  |  |  |  |  |  |
